# Effects of breed on preferential intake of hydroxychloride and sulfate sources of trace minerals in growing beef heifers

**DOI:** 10.1093/tas/txad130

**Published:** 2023-11-24

**Authors:** Isabella R T Souza, Philipe Moriel, Murillo H Barbosa, Maria E Rezende, Felipe Biazotto, Gian V A R Camargo, Karolina V Z Augusto, Marco A F Porcionato, Davi B Araujo, José L M Vasconcelos

**Affiliations:** Faculdade de Medicina Veterinária e Zootecnia, Universidade Estadual Paulista, Botucatu, SP 18618-970, Brazil; IFAS – Range Cattle Research and Education Center, University of Florida, Ona, FL 33865, USA; Faculdade de Medicina Veterinária e Zootecnia, Universidade Estadual Paulista, Botucatu, SP 18618-970, Brazil; Faculdade de Medicina Veterinária e Zootecnia, Universidade Estadual Paulista, Botucatu, SP 18618-970, Brazil; Faculdade de Medicina Veterinária e Zootecnia, Universidade Estadual Paulista, Botucatu, SP 18618-970, Brazil; Faculdade de Medicina Veterinária e Zootecnia, Universidade Estadual Paulista, Botucatu, SP 18618-970, Brazil; Trouw Nutrition Brazil, Campinas, SP 13080, Brazil; Trouw Nutrition Brazil, Campinas, SP 13080, Brazil; Nutreco Nederland, Selko Feed Additives, Amersfoort, 3811MH, The Netherlands; Faculdade de Medicina Veterinária e Zootecnia, Universidade Estadual Paulista, Botucatu, SP 18618-970, Brazil

**Keywords:** beef heifers, *Bos indicus*, hydroxychloride, sulfate, trace minerals

## Abstract

This study evaluated the effects of breed on voluntary preferential intake of two sources of Cu, Mn, and Zn when added to white salt-based trace mineral supplement (days 0 to 55; experiment 1) and protein supplement (days 56 to 112; experiment 2). On day 0, Nelore and ½ Angus × ½ Nelore heifers (*n* = 20/breed) were stratified by breed, body weight (BW = 347 ± 82 kg), and age (12 to 30 mo), and randomly allocated into 1 of the 40 drylot pens (1 heifer/pen). Both experiments were divided into washout (days 0 to 27 in experiment 1 and days 56 to 83 in experiment 2) and preferential intake periods (days 28 to 55 in experiment 1 and days 84 to 112 in experiment 2). During the respective preferential intake period, heifers were provided simultaneous free-choice access to sulfate (SUL) and hydroxychloride (HYD) sources of Cu, Mn, and Zn mixed into salt-based trace mineral supplements from days 28 to 55 (experiment 1) and then protein supplements from days 84 to 112 (experiment 2). Heifers were provided free-choice access to Tifton 85 (*Cynodon sp.*) hay from days 0 to 112. Effects of breed × source × day of the study were detected (*P* ≤ 0.05) for daily trace mineral intake from days 28 to 56 and days 84 to 112. Angus × Nelore heifers consumed a greater amount of SUL vs. HYD supplements on days 31 to 33 (*P* = 0.02) and HYD vs. SUL supplements on days 37 to 42 (*P* ≤ 0.02), whereas Nelore heifers consumed more HYD vs. SUL supplements on days 31 to 33 and 43 to 51 (*P* ≤ 0.05). Both breeds consumed (*P* ≤ 0.05) a greater amount of protein supplement containing HYD vs. SUL from days 84 to 112, but the differences in protein supplement intake increased (*P* ≤ 0.04) in greater magnitude for Nelore vs. Angus × Nelore heifers. Supplement intake coefficient of variation (CV) from days 28 to 41 and days 84 to 97 tended (*P* = 0.06) to be greater for Nelore vs. Angus × Nelore heifers. Effects of breed × source were detected (*P* = 0.02) for supplement intake CV from days 84 to 112. Intake CV of supplements added with HYD did not differ (*P* ≥ 0.40) between Nelore vs. Angus × Nelore heifers but was greater (*P* < 0.01) for Nelore vs. Angus × Nelore heifers fed SUL supplements. Overall, Nelore heifers had greater preferential intake for mineral and protein supplements containing hydroxychloride vs. sulfate sources compared to Angus × Nelore heifers. Hydroxychloride sources encouraged voluntary intake and reduced variation in supplement consumption compared to SUL sources of the same metals.

## Teaser

Nelore heifers had greater preferential intake for mineral and protein supplements fortified with hydroxychloride vs. sulfate sources of Cu, Zn, and Mn compared to Angus × Nelore heifers. Hydroxychloride sources encouraged voluntary intake and reduced variation in supplement consumption compared to sulfate sources of the same metals.

## Introduction

In tropical and subtropical regions, cattle typically graze year-round and trace mineral supplementation strategies are provided to maintain cowherd productivity. Free-choice, salt-based mineral supplements are often offered with the anticipation of adequate intake to offset nutrient deficiencies (Ranches et al., 2021a). However, relatively large fluctuations in the intake of free-choice trace mineral supplements among individuals decrease the efficiency of this supplementation strategy (Tait and Fisher, 1996; Greene, 2000). Fluctuation in mineral supplement consumption by grazing cattle can be affected by many factors, including season, geographical location, animal requirements, salt content of the water (McDowell, 1996), as well as trace mineral source (Caramalac et al., 2017; Ranches et al., 2021b) and breed (Ranches et al., 2021a).

Ionic salts, such as Zn sulfate, are highly soluble and dissociate quickly in the rumen, whereas hydroxychloride trace minerals are nearly insoluble in water (Spears et al., 2004), contributing to greater trace mineral supplement consumption compared to sulfate sources (Caramalac et al., 2017). Using a mineral feeder equipped with radio-frequency identification to evaluate the frequency of visits, Ranches et al. (2021a) demonstrated that there were no effects of breed on the number of visits during the morning and night periods, but Brahman cows made more visits to the mineral feeder in the afternoon period compared to Angus cows. Moreover, *Bos indicus*-influenced cattle displayed different grazing behavior, physiology (Cooke et al., 2020), and trace mineral metabolism (Ranches et al., 2021c) compared to *Bos taurus* breeds under similar management. Drawing conclusions based on the literature on other breeds is not prudent due to the described differences in intake behavior and mineral metabolism among cattle breeds. We hypothesized that Nelore (*B. indicus*) heifers would have a greater preference for hydroxychloride vs. sulfate sources of Cu, Mn, and Zn mixed into trace mineral supplementation compared to Angus × Nelore (*B. taurus-*influenced) heifers, but no breed effects would be detected when hydroxychloride and sulfate sources of Cu, Mn, and Zn are mixed into a protein supplement (dilution effect). Our objectives were to evaluate the effects of breed (Angus × Nelore vs. Nelore) on voluntary preferential intake and intake variability among individuals offered two sources (sulfate vs. hydroxychloride) of Cu, Mn, and Zn added to a white salt-based trace mineral supplement (experiment 1) and protein supplement (experiment 2).

## Materials and Methods

Experiments 1 and 2 were conducted at a commercial cow-calf operation (Santo Antônio located in São Manuel) (São Paulo, Brazil) from March until July 2023. All animals utilized in this experiment were cared for in accordance with acceptable practices and experimental protocols reviewed and approved by the Animal Ethical Use Committee from São Paulo State University (protocol #0304/2023).

### Experiment 1 (Trace Mineral Supplement; Days 0 to 55)

#### Animals and diets.

On day 0 of the study, 20 Nelore heifers and 20 ½ Angus × ½ Nelore heifers were stratified by breed, body weight (BW; on average 347 ± 82 kg), and age (on average 21 mo), and then randomly allocated into 1 of the 40 drylot pens (1 heifer/pen; 10 × 25 m). Heifers were provided free-choice access to Tifton 85 (*Cynodon sp.*) hay to ensure ad libitum consumption from days 0 to 55. Hay's nutritional value is shown in Table 1. Experiment 1 was divided into 2 periods of 28 d each: washout (days 0 to 27) and preferential intake (days 28 to 55).

**Table 1.** Nutritional composition^1^ (mean ± standard deviation) of Tifton 85 hay offered to Angus × Nelore and Nelore heifers from days 0 to 55 (experiment 1) and days 56 to 112 (experiment 2)

Item	Exp. 1 days 0 to 55	Exp. 2 days 84 to 112
Dry matter (DM), %	92.2 ± 2.3	94.5 ± 0.5
Non-fibrous carbohydrate, % of DM	5.6 ± 0.9	11.7 ± 4.3
Total digestible nutrients^2^, % of DM	50.1 ± 0.8	49.9 ± 2.9
Crude protein, % of DM	5.2 ± 0.8	5.4 ± 1.3
Neutral detergent fiber, % of DM	83.1 ± 1.7	81.3 ± 3.4
Acid detergent fiber, % of DM	49.2 ± 1.2	47.3 ± 3.5
Ether extract, % of DM	0.5 ± 0.3	0.6 ± 0.1
Cu, mg/kg	9 ± 4	6 ± 2.2
Mn, mg/kg	86 ± 29	65 ± 12
Zn, mg/kg	36 ± 12	41 ± 14

^1^Samples were collected from each pen every 14 d, pooled across pens within each day, and then sent to a commercial laboratory (Masterlab; Trouw nutrition, Arujá, São Paulo, Brazil) for wet chemistry analyses.

^2^Calculated as described by Weiss et al. (1992).

#### Washout period (days 0 to 27).

The washout period consisted of heifers provided free-choice access to hay and white salt for 28 d (Shaeffer et al., 2017; Guimaraes et al., 2021) to deplete mineral reserves of heifers (Spears et al., 2004; Shaeffer et al., 2017) and to acclimate heifers with stress of drylot entry and handling and avoid subsequent confounding effects on voluntary preferential intake of trace minerals. White salt and hay were provided daily at 0800 h and offered separately in covered, plastic feeders located at ground level and at the center of each pen.

#### Preferential intake period (days 28 to 55).

Preferential intake period consisted of heifers provided simultaneous free-choice access white salt-based trace mineral supplements containing sulfate (**SUL**) or hydroxychloride (**HYD**; Selko IntelliBond^®^, Selko Feed Additives) sources of Cu, Mn, and Zn for 28 d (days 28 to 55). The 28-d period of supplementation of SUL and HYD utilized herein was selected based on previous studies demonstrating that 21 d of supplementation of Zn hydroxychloride was sufficient to alter the fecal microbiome (Wenner et al., 2022) and alter the intestinal morphology and initiate a systemic inflammatory response compared to Zn sulfate supplementation (Horst et al., 2020). Both sources of Cu, Mn, and Zn were mixed into their respective white salt-based trace mineral supplement containing all macro and trace minerals, except for Cu, Mn, and Zn, and offered in a loose meal form. Each supplement was prepared by Trouw Nutrition following their production guidelines to avoid cross-contamination and was delivered at the research site in a single batch of multiple 30-kg weatherproof bags. Ingredient composition of each supplement is proprietary to Trouw Nutrition (Campinas, São Paulo, Brazil), but their respective chemical composition is shown in Table 2. Heifers were offered 130 g/day of each respective supplement. Both supplements were formulated to achieve a target intake of 50 to 100 g/day and provide a minimum of 1,350, 1,040, and 5,000 mg of Cu, Mn, and Zn per kg of supplement dry matter (DM), which is greater than the Cu, Mn, and Zn requirements of growing beef calves (NASEM, 2016). Hay and each respective trace mineral supplement were provided daily at 0800 h and offered separately in covered, plastic feeders located at ground level and at the center of each pen (Caramalac et al., 2017). In addition, the position of each respective plastic feeder of each supplement type (SUL and HYD) was swapped every 3 d to avoid any potential influence of location on daily intake of each trace mineral source.

**Table 2.** Nutritional composition^1^ (mean ± standard deviation) of mineral and protein supplements^2^ offered to Angus × Nelore and Nelore heifers from days 0 to 55 (experiment 1) and days 56 to 112 (experiment 2), respectively

	Experiment 1 (days 28 to 55)		Experiment 2 (days 84 to 112)	
Item	SUL	HYD	SUL	HYD
Dry matter (DM), %	85 ± 0.7	86 ± 0.7	94 ± 0.3	94 ± 0.3
Total digestible nutrients, %	-	-	42 ± 1.8	42 ± 2.0
Crude protein, %	-	-	47 ± 8.3	45 ± 1.5
Cu, mg/kg	1388 ± 276	1301 ± 264	220 ± 25	196 ± 33
Mn, mg/kg	1499 ± 155	1284 ± 143	162 ± 14	147 ± 5
Zn, mg/kg	5199 ± 1187	5565 ± 665	795 ± 24	752 ± 85

^1^Samples were collected prior to morning supplementation every 14 d from days 28 to 55 (experiment 1) and days 84 to 112 (experiment 2) and then sent to a commercial laboratory (Masterlab; Trouw nutrition, Arujá, São Paulo, Brazil) for wet chemistry analyses.

^2^Experiment 1 = white salt-based trace mineral supplements (BellNutri, Trouw Nutrition, Campinas, São Paulo, Brazil) added with SUL and HYD sources of Cu, Mn, and Zn were both formulated for a target intake of 50 to 100 g/day and to provide a minimum of 14% Ca, 8% P, 13% Na, 1% Mg, 4% S, 80 mg/kg Co, 100 mg/kg I, and 26 mg/kg Se, in addition to the amount of Cu, Mn, and Zn described above. Experiment 2 = protein supplements (Lambisk VS, Trouw Nutrition, Campinas, São Paulo, Brazil) added with SUL and HYD sources of Cu, Mn, and Zn were both formulated for a target intake of 0.1% to 0.2% of body weight (DM basis) and to provide a minimum of 5% Ca, 1.5% P, 3% Na, 1.5% S, 2,000 mg/kg Mg, 15 mg/kg Co, 19 mg/kg I, and 5 mg/kg Se, in addition to the amount of Cu, Mn, and Zn described above.

### Experiment 2 (Protein Supplement; Days 56 to 112)

#### Animals and diets.

Experiment 2 was initiated on day 56 and was divided into two periods of 28 d each: washout (days 56 to 83) and protein supplement preferential intake (days 84 to 112). Heifers were provided free-choice access to Tifton 85 (*Cynodon sp.*) hay to ensure ad libitum consumption from days 56 to 112. Hay's nutritional value is shown in Table 1.

#### Washout period (days 56 to 83).

The washout period consisted of heifers provided free-choice access to hay and white salt for 28 d (Shaeffer et al., 2017; Guimaraes et al., 2021) to deplete mineral reserves of heifers (Spears et al., 2004; Shaeffer et al., 2017) and to minimize any potential confounding effects of experiment 1 on voluntary trace mineral intake during experiment 2. White salt and hay were provided daily at 0800 h and offered separately in covered, plastic feeders located at ground level and at the center of each pen. Heifers remained on their respective pen which was assigned on day 0.

#### Protein supplement preferential intake (days 84 to 112).

The protein supplement preferential intake period consisted of heifers provided simultaneous free-choice access to protein supplementation containing sulfate (**SUL**) or hydroxychloride (**HYD**; Selko IntelliBond, Selko Feed Additives) sources of Cu, Mn, and Zn for 28 d (days 84 to 112). Both sources of Cu, Mn, and Zn were mixed into their respective loose meal protein supplement, which contained all macro and trace minerals, except for Cu, Mn, and Zn. Each protein supplement was prepared by Trouw Nutrition following their production guidelines to avoid cross-contamination and was delivered at the research site in a single batch of multiple 30-kg weatherproof bags. Ingredient composition of each protein supplement added with SUL or HYD sources is proprietary to Trouw Nutrition (Campinas, São Paulo, Brazil), but their respective chemical composition is shown in Table 2. Heifers were offered 1.8 to 3 kg/day of each respective supplement to ensure free-choice access to each mineral source. Both supplements were formulated to achieve a target intake of 0.10% to 0.20% of BW (DM basis) and provide a minimum of 260, 200, and 960 mg of Cu, Mn, and Zn per kg of supplement DM. Hay and each respective supplement were offered separately in covered, plastic tubs located at ground level and at the center of each pen (Caramalac et al., 2017). In addition, the position of each respective plastic feeder of each supplement type (SUL and HYD) was swapped every 3 d to avoid any potential influence of location on daily intake of each protein supplement.

### Data Collection

Individual heifer-shrunk BW were collected every 28 d from days 0 to 112, following 12 h of feed withdrawal. In both experiments, consumption (g/day) of SUL and HYD supplements was calculated daily by subtracting the as-fed amount refused from the as-fed amount offered of each supplement in the previous day from days 28 to 55 (experiment 1) and days 84 to 112 (experiment 2). Hand samples of each supplement were collected daily from each pen and then dried for 96 h at 50 °C in forced-air ovens to assess the respective DM concentration of each sample. Supplement DM intake (g/day) was calculated by multiplying the respective DM concentration of each supplement by the as-fed amount consumed on that same day, and then averaged and reported every 3 d from days 28 to 55 (experiment 1) and days 84 to 112 (experiment 2). In both experiments, intake of each respective supplement source was also reported as % of total supplement intake from days 28 to 56 and days 84 to 112, and as g of supplement per 100 kg of BW using the average BW from days 28 to 56 and days 84 to 112 for experiments 1 and 2, respectively. Samples of white salt-based trace mineral (experiment 1) and protein (experiment 2) supplements were collected prior to morning supplementation every 14 d from days 28 to 55 (experiment 1) and days 84 to 112 (experiment 2), and then sent to a commercial laboratory (Masterlab; Trouw nutrition, Arujá, São Paulo, Brazil) for wet chemistry analyses.

In both experiments, hay samples were collected from each pen every 14 d (days 0 to 56 in experiment 1 and days 56 to 112 in experiment 2), pooled across pens within each day, and then sent to a commercial laboratory (Masterlab; Trouw nutrition, Arujá, São Paulo, Brazil) to assess the nutritional composition. Samples were analyzed by wet chemistry procedures for concentrations of crude protein (method 984.13; AOAC, 2006), acid detergent fiber (method 973.18 modified for use in an Ankom 200 fiber analyzer, Ankom Technology Corp., Fairport, NY; AOAC, 2006), neutral detergent fiber (modified for Ankom 200 fiber analyzer, Ankom Technology Corp.), and Cu, Mn, and Zn (method 985.30; AOAC, 2006).

### Statistical Analyses

Heifer was considered the experimental unit and included as random effect in all statistical analyses. Data were analyzed as a complete randomized design study using the MIXED procedure of SAS (SAS Institute Inc., Cary, NC, USA, version 9.4) with Satterthwaite approximation to determine the denominator degrees of freedom for the test of fixed effects. In both experiments, heifer average daily gain (ADG) was tested for fixed effects of breed, whereas heifer BW were analyzed as repeated measures and tested for fixed effects of breed, day of the study, and resulting interaction. In both experiments, heifer supplement DM intake (% of total, g/100 kg of BW, and coefficient of variation, CV) were analyzed as repeated measures and tested for fixed effects of breed, mineral source (SUL vs. HYD), day of the study, and all resulting interactions. Compound symmetry was the selected covariance structure for each repeated measures analysis because it generated the lowest Akaike information criterion. Heifer BW on day 0 and age were initially included as covariates in all statistical analyses but removed from the model because *P* > 0.10. All results were reported as least-square means. Data were separated using PDIFF if a significant F-test was detected. Significance was set at *P* ≤ 0.05, and tendencies were noted if *P* > 0.05 and ≤ 0.10.

## Results

### Experiment 1

Effects of breed × day of the study were detected (*P *= 0.04) for heifer BW, which did not differ on days 0 and 56 (*P* ≥ 0.19) but tended (*P* = 0.07) to be greater for Angus × Nelore vs. Nelore heifers on day 28 (Table 3). Heifer ADG from days 0 to 28 was greater (*P* < 0.01) for Angus × Nelore vs. Nelore heifers, whereas ADG from days 28 to 56 and 0 to 56 did not differ (*P* ≥ 0.11) between breeds (Table 3).

**Table 3.** Body weight (**BW**) and average daily gain (**ADG**) of Angus × Nelore (**AN**) and Nelore (**NE**) heifers provided free-choice access to sulfate (**SUL**) and hydroxychloride (**HYD**) sources of Cu, Mn, and Zn mixed into a white salt-based trace mineral supplement from days 28 to 55 (experiment 1) or mixed into a protein supplement from days 84 to 112 (experiment 2).^1^

	Breed			
Item`	AN	NE	SEM	*P*-value^2^
Experiment 1				
BW^3^, kg				
Day 0	350	349	3.2	0.82
Day 28	372	362	3.2	0.07
Day 56	376	368	3.2	0.19
ADG, kg/day				
Days 0 to 28	0.79	0.48	0.057	<0.01
Days 28 to 56	0.13	0.23	0.095	0.44
Day 0 to 56	0.46	0.35	0.044	0.11
Experiment 2				
BW, kg				
Day 56	376	368	6.5	0.19
Day 84	380	357	6.5	0.05
Day 112	382	365	6.5	0.14
ADG, kg/day				
Days 56 to 84^3^	0.11	-0.38	0.134	0.07
Days 84 to 112	0.31	0.05	0.216	0.50
Days 56 to 112^3^	0.21	-0.17	0.104	0.05

^1^In both experiments, heifers were provided free-choice access to ground hay throughout the entire study. Heifers were also assigned to a washout period of 28 d and provided free-choice access to white salt only from days 0 to 28 (experiment 1) and days 56 to 84 (experiment 2).

^2^ *P*-value for the comparison of breed within day of the study for heifer BW or *P*-value for the effects of breed for heifer ADG.

^3^Covariate-adjusted for heifer BW on day 0 for experiment 1 and day 56 for experiment 2 (*P* ≤ 0.02).

**Figure 1.**
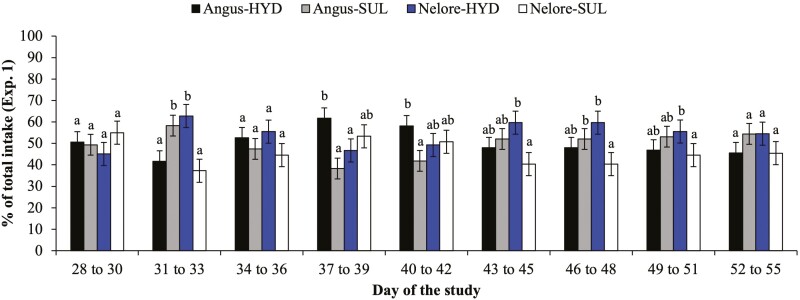
Average daily supplement intake (% of total intake; experiment 1) of Angus × Nelore and Nelore heifers provided simultaneous free-choice access to salt-based trace mineral supplementation containing sulfate (**SUL**) or hydroxychloride (**HYD**) sources of Cu, Mn, and Zn from days 28 to 55. Effects of breed × source × day of the study were detected (*P *< 0.0001) for trace mineral intake, calculated as % of total intake. Angus × Nelore heifers consumed more SUL vs. HYD supplements on days 31 to 33 (*P* = 0.02) and more HYD vs. SUL supplements on days 37 to 42 (*P* ≤ 0.02). Nelore heifers consumed more HYD vs. SUL supplements on days 31 to 33 and 43 to 51 (*P* ≤ 0.05). ^ac^*P* ≤ 0.05.

Effects of breed × source × day of the study were detected (*P* ≤ 0.05) for daily trace mineral intake from days 28 to 56 calculated as % of total intake (Figure 1) and g per 100 kg of BW (Table 4). In Angus × Nelore heifers, daily trace mineral intake (% of total) of SUL and HYD supplements did not differ (*P* ≥ 0.22) on days 28 to 30, 34 to 36, and 43 to 55 (Figure 1). However, Angus × Nelore heifers consumed a greater amount of SUL vs. HYD supplements on days 31 to 33 (*P* = 0.02) and a greater amount of HYD vs. SUL supplements on days 37 to 42 (*P* ≤ 0.02; Figure 1). In Nelore heifers, daily trace mineral intake (% of total) of SUL and HYD supplements did not differ (*P* ≥ 0.16) on days 28 to 30, 34 to 42, and 52 to 55 (Figure 1). However, Nelore heifers consumed a greater daily amount of HYD vs. SUL supplements on days 31 to 33 and 43 to 51 (*P* ≤ 0.05; Figure 1). In Angus × Nelore heifers, daily trace mineral intake (g per 100 kg of BW) of SUL and HYD supplements did not differ (*P* ≥ 0.11) on days 28 to 30, 34 to 36, and 40 to 55 (Table 4). However, Angus × Nelore heifers consumed a greater amount of SUL vs. HYD supplements on days 31 to 33 (*P* = 0.05) and a greater amount of HYD vs. SUL supplements on days 37 to 39 (*P* = 0.04; Table 4). In Nelore heifers, daily trace mineral intake (g per 100 kg of BW) of SUL and HYD supplements did not differ (*P* ≥ 0.69) on days 28 to 30 and 34 to 42 (Table 4). However, Nelore heifers consumed a greater amount of HYD vs. SUL supplements on days 31 to 33 and 43 to 55 (*P* ≤ 0.05; Table 4). Average supplement intake (g per 100 kg of BW) from day 28 to 55 did not differ (*P* ≥ 0.13) between Nelore and Angus × Nelore heifers when supplements contained HYD (9.0 ± 0.7 vs. 9.0 ± 0.7 g per 100 kg of BW for Nelore and Angus × Nelore heifers, respectively) or SUL sources (7.1 ± 0.7 vs. 8.6 ± 0.7 g per 100 kg of BW for Nelore and Angus × Nelore heifers, respectively).

**Table 4.** Daily supplement intake (g per 100 kg of body weight [BW])^1^ of Angus × Nelore (**AN**) and Nelore (**NE**) heifers provided free-choice access to sulfate (**SUL**) and hydroxychloride (**HYD**) sources of Cu, Mn, and Zn mixed into a white salt-based trace mineral supplement from days 28 to 55 (experiment 1) or mixed into a protein supplement from days 84 to 112 (experiment 2)^2^

	Day of the study										
Item	28 to 30	31 to 33	34 to 36	37 to 39	40 to 42	43 to 45	46 to 48	49 to 51	52 to 55	SEM	*P* ^3^
*Experiment 1*											
AN-HYD	7^a^	8^b^	11^a^	13^b^	12^a^	8^a,b^	8^a,b^	8^a,b^	8^b^	1.4	0.05
AN-SUL	6^a^	12^c^	11^a^	10^a^	9^a^	7^a,b^	7^a,b^	9^a,b^	8^b^	1.4	
NE-HYD	7^a^	7^b^	10^a^	10^a^	9^a^	10^b^	10^b^	11^b^	8^b^	1.5	
NE-SUL	9^a^	3^a^	8^a^	11^a,b^	9^a^	6^a^	6^a^	8^a^	4^a^	1.5	

	84 to 86	87 to 89	90 to 92	93 to 95	96 to 98	99 to 101	102 to 104	105 to 107	108 to 112		
*Experiment 2*											
AN-HYD	154^d^	159^c^	109^c^	173^d^	151^c^	152^c^	98^c^	144^c^	95^b^	11.3	0.05
AN-SUL	90^b^	91^b^	61^b^	88^b^	68^b^	90^b^	57^a,b^	69^b^	39^a^	11.3	
NE-HYD	110^c^	157^c^	106^c^	137^c^	97^b^	117^c^	83^b,c^	128^c^	96^b^	12.2	
NE-SUL	31^a^	36^a^	18^a^	45^a^	27^a^	42^a^	30^a^	22^a^	15^a^	12.2	

^a–d^Within day of the study, means without a common superscript differ (*P* ≤ 0.05).

^1^Calculated by dividing the respective 3-d average DM supplement intake (g/d) by the average BW from days 28 to 56 (experiment 1) and days 84 to 112 (experiment 2), and then multiplied by 100.

^2^In both experiments, heifers were provided free-choice access to ground hay throughout the entire study. Heifers were also assigned to a washout period of 28 d and provided free-choice access to white salt only from days 0 to 28 (experiment 1) and days 56 to 84 (experiment 2).

^3^ *P*-value for the effects of breed × source × day of the study.

Effects of breed × source and source were not detected (*P* ≥ 0.28) for supplement intake CV (Table 5). Effects of breed tended (*P* = 0.06) to be detected for supplement intake CV from days 28 to 41, which tended (*P* = 0.06) to be greater for Nelore vs. Angus × Nelore heifers (70 ± 4.1% vs. 57 ± 4.1%, respectively).

**Table 5.** Supplement intake coefficient of variation^1^ of Angus × Nelore (**AN**) and Nelore (**NE**) heifers provided free-choice access to sulfate (**SUL**) and hydroxychloride (**HYD**) sources of Cu, Mn, and Zn mixed into a white salt-based trace mineral supplement from days 28 to 55 (experiment 1) or mixed into a protein supplement from days 84 to 112 (experiment 2)^2^

	Breed × Source					*P*-value		
Item	AN-HYD	AN-SUL	NE-HYD	NE-SUL	SEM	Breed × source	Breed	Source
*Experiment 1*								
Days 28 to 41	57	60	70	70	7.2	0.76	0.06	0.77
Days 42 to 56	60	50	56	50	7.9	0.83	0.81	0.28
Days 28 to 56	70	75	75	80	6.1	0.96	0.40	0.36
*Experiment 2*								
Days 84 to 97	66	104	71	133	9.7	0.21	0.06	<0.01
Days 98 to 112	71^a^	119^c^	57^a^	163^d^	11.8	0.01	0.22	<0.01
Days 84 to 112	69^a^	111^b^	65^a^	153^c^	9.7	0.02	0.05	<0.01

^a–d^Within day of the study, means without a common superscript differ (*P* ≤ 0.05).

^1^Calculated by dividing the standard deviation of daily DM supplement intake (g/d) by the average daily supplement intake from days 28 to 56 (experiment 1) and days 84 to 112 (experiment 2), and then multiplied by 100.

### Experiment 2

Effects of breed × day of the study were detected (*P *= 0.04) for heifer BW, which was greater (*P* = 0.05) for Angus × Nelore vs. Nelore heifers on day 84 and did not differ (*P* ≥ 0.14) on days 56 and 112. Heifer ADG from days 56 to 84 tended (*P* = 0.07) to be greater for Angus × Nelore vs. Nelore heifers, did not differ (*P* = 0.50) between breeds from days 84 to 112, and was greater (*P* = 0.05) for Angus × Nelore vs. Nelore heifers from days 56 to 112 (Table 3).

**Figure 2.**
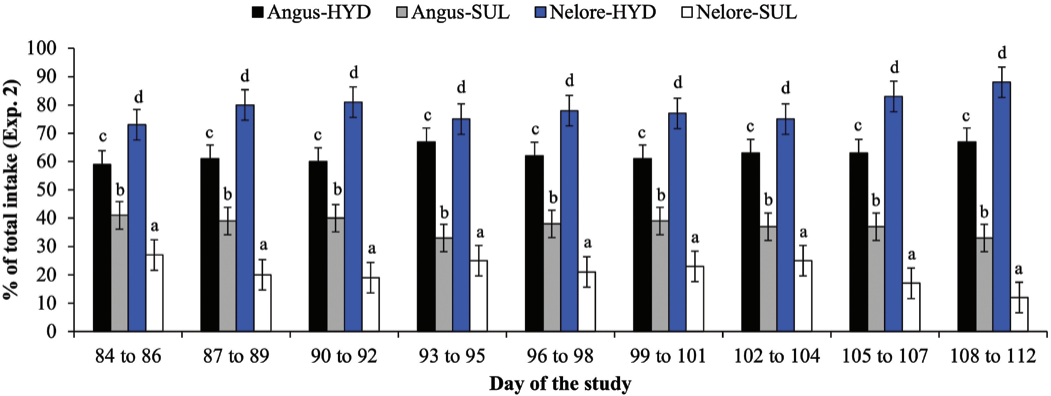
Average daily supplement intake (% of total intake; experiment 2) of Angus × Nelore and Nelore heifers provided simultaneous free-choice access to protein supplementation containing sulfate (**SUL**) or hydroxychloride (**HYD**) sources of Cu, Mn, and Zn mixed from days 84 to 112. Both breeds consumed (*P* ≤ 0.05) a greater daily amount of protein supplement containing HYD vs. SUL from days 84 to 112, but the differences in daily protein supplement intake between both mineral sources gradually increased over time (*P* ≤ 0.04) and in greater magnitude for Nelore vs. Angus × Nelore heifers. ^ac^*P* ≤ 0.05.

Effects of breed × source × day of the study were detected (*P* ≤ 0.05) for daily protein supplement intake from days 84 to 112, calculated as % of total intake (Figure 2) and g per 100 kg of BW (Table 4). Both breeds consumed (*P* ≤ 0.05) a greater amount of protein supplement containing HYD vs. SUL from days 84 to 112, but the differences in protein supplement intake (% of total and g per 100 kg of BW) between both mineral sources gradually increased over time (*P* ≤ 0.04) and in greater magnitude for Nelore vs. Angus × Nelore heifers (Figure 2 and Table 4). Average supplement intake (g per 100 kg of BW) from days 84 to 112 added with HYD did not differ (*P* = 0.26) between Nelore and Angus × Nelore heifers (115 ± 13.5 vs. 137 ± 13.5 g per 100 kg of BW, respectively), whereas average supplement intake added with SUL was greater (*P* = 0.03) for Angus × Nelore vs. Nelore heifers (73 ± 13.5 vs. 30 ± 13.5 g per 100 kg of BW, respectively).

Effects of breed × source were not detected (*P* ≥ 0.21) for supplement intake CV from days 84 to 97 (Table 5). Effects of breed tended (*P* = 0.06) to be detected and effects of source were detected (*P* < 0.01) for supplement intake CV from days 84 to 97, which tended (*P* = 0.06) to be greater for Nelore vs. Angus × Nelore heifers (102 ± 6.8% vs. 85 ± 6.3%, respectively) and was greater (*P* < 0.01) for SUL vs. HYD mineral sources (119 ± 6.6% vs. 68 ± 6.6%, respectively). Effects of breed × source were detected (*P* ≤ 0.02) for supplement intake CV from days 98 to 112 and 84 to 112. Intake CV of supplements added with HYD did not differ (*P* ≥ 0.40) between Nelore vs. Angus × Nelore heifers, whereas intake CV of supplements added with SUL was greater (*P* < 0.01) for Nelore vs. Angus × Nelore heifers (Table 5).

## Discussion

Variation in the intake of free-choice supplements is a common obstacle to decreasing the efficiency of mineral supplementation (Greene, 2000). Fluctuation in mineral supplement consumption by grazing beef cattle can be affected by season, dietary energy, and protein, individual mineral requirements, product palatability, salt content of the water, soil properties, geographical location (McDowell, 1996), forage quality and quantity (McClain et al., 2020), location of mineral feeder, and cattle subspecies (Ranches et al., 2021a, b, c). Experiments 1 and 2 are the first set of multiple sequential studies conducted by our group evaluating the impacts of breed on beef cattle performance grazing warm-season grasses and supplemented with different mineral sources in subtropical environments. Daily monitoring of hay DM intake was not possible in both experiments, so our initial step was to evaluate any breed-induced differences in voluntary preferential intake of supplements (mineral- and protein-based supplements) when heifers were provided simultaneous access to SUL or HYD sources of Cu, Mn, and Zn. Any interpretation for the differences in growth performance detected between breeds in experiments. 1 and 2 are difficult to expand without knowing the exact hay DM intake of each breed and because heifers were provided simultaneous access to both treatments. Nonetheless, ADG differed between breeds during both washout periods and did not differ between breeds when HYD and SUL supplements were provided (days 28 to 56 and days 84 to 112), which perhaps occurred due to the differences in mineral metabolism between breeds (Ranches et al., 2021c) allowing Nelore heifers to maintain a relatively similar growth performance despite the less total supplement intake (SUL + HYD) in experiment 2 compared to Angus × Nelore heifers. Breed- and mineral-source-induced differences in growth performance and mineral status of Nelore vs. Angus × Nelore heifers will be evaluated in subsequent studies providing access to only 1 or both mineral sources and with daily monitoring of forage DM intake.

Hydroxychloride trace minerals are virtually insoluble in water or weak acidic pH but are solubilized under acidic conditions in the abomasum (Spears et al., 2004), whereas minerals in sulfate form are almost completely soluble in water (Spears et al., 2004). Growing beef calves had an aversion to consuming mineral-concentrated feeds (Moriel and Arthington, 2013) and preferred supplements added with HYD vs. SUL mineral sources (Caramalac et al., 2017; Ranches et al., 2021b), which is likely attributed to a potential “metallic taste” resulting from soluble metal ingredients delivered in sulfate form. In addition, it has been shown that Brahman (*B. indicus*) cows had higher frequency of visits to the mineral feeder compared to Angus (*B. taurus*) cows (Ranches et al., 2021a). Hence, we hypothesized that Nelore heifers would have a greater preference for HYD vs. SUL mineral sources when both mineral sources were added into a white salt-based mineral supplement (experiment 1), whereas no breed-induced differences in supplement preferential intake would be detected when HYD and SUL elements were added into a protein supplement due to a dilution effect (experiment 2).

In agreement with our hypothesis, Angus × Nelore heifers in experiment 1 alternated between SUL and HYD mineral supplements during the first 14 d of supplementation but demonstrated no preference for any mineral source after 14 d of supplementation (Figure 1). In contrast, Nelore heifers demonstrated a greater preference for HYD vs. SUL mineral supplements during most of the supplementation period. Contrary to our hypothesis, both breeds had a greater preference for HYD vs. SUL protein supplements in experiment 2, with Angus × Nelore heifers maintaining a relatively constant preference for HYD vs. SUL protein supplements, whereas Nelore heifers gradually increased their preference for protein supplements added with HYD vs. SUL from days 84 to 112 (Figure 2). It is plausible that: (1) the greater preferential intake of supplements containing HYD vs. SUL sources was because of the high solubility and “metallic” taste aversion of sulfate minerals, regardless of supplement amount and type (mineral vs. protein supplements), and (2) Nelore heifers exhibit a greater aversion to metallic taste of SUL supplements compared to Angus × Nelore heifers. Moreover, the results observed in experiment 2 were unexpected, and perhaps can be explained by the fact that the same heifers were used in both experiments and the previous exposure to HYD supplementation in experiment 1 may have conditioned or trained heifers to choose the same mineral source compared to SUL supplementation, despite the implementation of a washout period of 28 d between experiments and the frequent swapping of feeder location of each respective mineral source. Nonetheless, the magnitude of preferential intake for protein supplements added with HYD in experiment 2 (>80% of total supplement intake for Nelore heifers and 60% to 70% for Angus × Nelore heifers) was similar to those reported by Caramalac et al. (2017). Early weaned, Brangus (*B. indicus*-influenced) crossbred calves almost exclusively selected hydroxychloride vs. sulfate or organic sources mixed into a loose meal, citrus pulp-based supplement (83%, 10%, and 7% of total supplement intake for hydroxychloride, sulfate, and organic sources, respectively; Caramalac et al., 2017), whereas early weaned, Brangus crossbred calves also preferred hydroxychloride vs. organic and sulfate sources but in less magnitude (39%, 30%, and 31% of total supplement intake for hydroxychloride, organic, and sulfate, respectively) when added into sugarcane molasses-based protein blocks (Ranches et al., 2021b). Therefore, supplement amount, ingredient composition, and delivery form (block vs. loose meal) and breed may impact the magnitude of preferential intake for HYD vs. SUL supplements and must be taken into consideration when formulating supplements for beef cattle. These results also reinforce the need for similar evaluations of supplement preferential intake using different animal categories and supplementation strategies.

Daily supplement preferential intake was also calculated as g per 100 kg of BW to account for any differences in BW among heifers. As expected, fluctuations and outcomes to daily supplement intake calculated as g per 100 kg of BW mimicked the results reported as % of total supplement intake in both experiments. More importantly, average total mineral supplement intake (SUL + HYD) in experiment 1 achieved the target intake for Angus × Nelore heifers (intake ranged from 46 to 80 g/day) and was either below or within the lower end of target intake for Nelore heifers (35 to 77 g/day), which was primarily due to the reduced SUL intake of Nelore vs. Angus × Nelore heifers during the final 14 d of supplementation in experiment 1. In contrast, average intake of protein supplements (SUL + HYD) from days 84 to 112 in experiment 2 represented ~0.21% and 0.14% of BW for Angus × Nelore and Nelore heifers, respectively, which were 40% to 100% greater than the minimum formulated target intake (0.10% of BW) and perhaps explained by the relatively small pasture size, heifer age, acclimation to pens, and low nutritional value of forage provided to heifers encouraging protein supplement intake (McDowell, 1996; McClain et al., 2020). More importantly, average protein supplement intake of Angus × Nelore heifers was ~0.14% and 0.07% of BW for HYD and SUL sources, whereas protein supplement intake of Nelore heifers was ~0.11% and 0.03% of BW for HYD and SUL sources, respectively. Overall, protein supplements added with HYD sources allowed heifers to achieve or surpass the target supplement intake, whereas adding SUL sources into protein supplementation prevented heifers from achieving their target intake, regardless of breed.

Salt-limited supplements are a convenient delivery method that can increase low-quality forage intake and improve cattle performance (McCollum and Horn, 1990). However, intake variation among individuals remains as a concern (Tait and Fisher, 1996; Bowman and Sowell, 1997), which could lead to either mineral deficiency or supplement wastage and antagonistic effects among mineral elements present in both supplement and forage consumed (McDowell, 1996). Therefore, we also calculated the CV for each mineral source in both experiments using the average daily supplement intake of each heifer from days 28 to 55 (experiment 1) and days 84 to 112 (experiment 2). Nelore heifers tended to have a slightly greater CV for supplement intake during the first 14 d of supplementation in both experiments compared to Angus × Nelore heifers. Although not evaluated herein, this greater CV for mineral and protein supplement intake of Nelore heifers may be associated with a greater frequency of visits to the mineral feeder compared to Angus × Nelore heifers, as previously reported for Brahman vs. Angus cows (Ranches et al., 2021a). In experiment 2, protein supplementation added with HYD source had similar CV for supplement intake between Nelore and Angus × Nelore heifers. In contrast, CV for supplement intake of protein supplementation added with SUL was significantly greater compared to HYD supplements and was greater for Nelore vs. Angus × Nelore heifers. The greater variation of protein supplement intake added with SUL vs. HYD sources are likely due to the aversion effects of adding SUL elements, as described above, whereas the greater variation of protein supplement intake of Nelore vs. Angus × Nelore heifers may be possibly due to a greater aversion to SUL minerals in Nelore vs. Angus × Nelore heifers.

In summary, Nelore heifers consuming warm-season forage had greater preferential intake for mineral and protein supplements added with hydroxychloride vs. sulfate sources of Cu, Mn, and Zn compared to Angus × Nelore heifers. In addition, hydroxychloride sources of Cu, Zn, and Mn addressed the issue of suboptimal voluntary intake, leading to greater supplement intake after 14 d of supplementation, and reduced initial and long-term variation in supplement consumption compared to sulfate sources of the same metals.
